# Thermo-Physiological Comfort Properties of Sportswear with Different Combination of Inner and Outer Layers

**DOI:** 10.3390/ma14226863

**Published:** 2021-11-14

**Authors:** Desalegn Atalie, Pavla Tesinova, Melkie Getnet Tadesse, Eyasu Ferede, Ionuț Dulgheriu, Emil Loghin

**Affiliations:** 1 Ethiopian Institute of Textile and Fashion Technology, Bahir Dar University, Bahir Dar 1037, Ethiopia; desalegnatalie@gmail.com (D.A.); eyasuferede1982@gmail.com (E.F.); 2 Department of Textile Evaluation, Technical University of Liberec, 46117 Liberec, Czech Republic; pavla.tesionova@tul.cz; 3 Faculty of Industrial Design and Business Administration, Gheorghe Asachi Technical University of Iasi, 53, D. Mangeron Blv., 700050 Iasi, Romania; ionut.dulgheriu@academic.tuiasi.ro

**Keywords:** sportswear, thermal resistance, thermal conductivity, thermal absorptivity, peak heat flow density ratio, stationary heat flow density, water vapor permeability, bi-layered sportswear

## Abstract

Consumers expect high-performance functionality from sportswear. To meet athletic and leisure-time activity requirements, further research needs to be carried out. Sportswear layers and their specific thermal qualities, as well as the set and air layer between materials, are all important factors in sports clothing. This research aims to examine the thermal properties of sports fabrics, and how they are affected by structure parameters and maintained with different layers. Three inner and four outer layers of fabric were used to make 12 sets of sportswear in this study. Before the combination of outer and inner layers, thermal properties were measured for each individual layer. Finally, the thermal resistance, thermal conductivity, thermal absorptivity, peak heat flow density ratio, stationary heat flow density, and water vapor permeability of bi-layered sportswear were evaluated and analyzed. The findings show that sportswear made from a 60% cotton/30% polyester/10% elastane inner layer and a 100% polyester outer layer had the maximum thermal resistance of 61.16 (×10^3^ K·m^2^ W^−1^). This performance was followed by the sample made from a 90% polyester/10% elastane inner layer and a 100% polyester outer layer, and the sample composed of a 100% elastane inner layer and a 100% polyester outer layer, which achieved a thermal resistance value of 60.41 and 59.41 (×10^3^ K·m^2^ W^−1^), respectively. These results can be explained by the fact that thicker textiles have a higher thermal resistance. This high-thermal-resistance sportswear fabric is appropriate for the winter season. Sportswear with a 90% polyester/10% elastane inner layer had worse water vapor resistance than sportswear with a 60% cotton/30% polyester/10% elastane and a 100% elastane layer. Therefore, these sports clothes have a higher breathability and can provide the wearers with very good comfort. According to the findings, water vapor permeability of bi-layered sportswear is influenced by geometric characteristics and material properties.

## 1. Introduction

Due to an increase in interest in the indoor–outdoor sports and outdoor leisure hobbies, there has been a considerable growth in sportswear consumption in recent years. More interest in sports has been fueled by more leisure time, increased health awareness, increased indoor and outdoor recreation facilities, and the advent of well-designed functional apparel [1]. The use of active sporting materials has increased in recent years, allowing them to serve several activities while maintaining comfort. The use of cutting-edge textile science and technology in the production of sports and leisurewear materials is advancing every day in order to meet the demands for the improvement of performance in athletic and leisure activities [2]. Many sports activities have different clothing expectations, and the requirements for sportswear change according to the weather. For example, cold-season sports apparel must provide adequate thermal insulation as well as weather protection [3]. In dry weather, windproof clothing may suffice, but in wet weather, a waterproof outer layer is required. Low heat insulation qualities and high air permeability are expected from clothes worn in hot weather conditions to reduce heat stress. Water repellency properties are preferable to air permeability blocking in hot and rainy weather conditions [4].

Much research has focused on bi-layer structures to achieve a high level of comfort [5,6,7,8,9,10]. This is because the performance of layered fabrics in thermo-physiological regulation is better than that of single-layer textile structures [11,12,13,14]. Several researchers have investigated the influence of fiber fineness and cross-sectional shapes [15,16] as well as the effects of fiber type, stitch type [17,18], yarn type [19], fabric structure [20,21,22,23,24], bi-layer fabrics [25,26,27], multi-layer sportswear [28,29], blended fabric [30,31], and 2D and 3D designs of sportswear [17,32,33,34].

Researchers reported that bilayer fabrics (knitted/woven) can be used as moisture-management fabrics without any additional treatments [30,35]. Mbise et al. found that the hydrostatic pressure difference between the two layers of spacer fabric is one of the factors affecting moisture transfer [8]. A studies on the effect of yarn composition and knitting structure on bi-layer knitted fabrics [36]; a comparative study of eri-silk, wool, and bamboo knitted fabrics [37]; and a study of 100% eri-silk for active sportswear applications [38] have previously been conducted. The air permeability, water vapor permeability, thermal conductivity, wicking, and drying ability of bi-layer knitted fabric made up of polypropylene as the inner layer and modal as the outer layer with one tuck point of repeat were found to be higher when compared to other bi-layer, plated, and single jersey structures [6].

**Figure 1:**
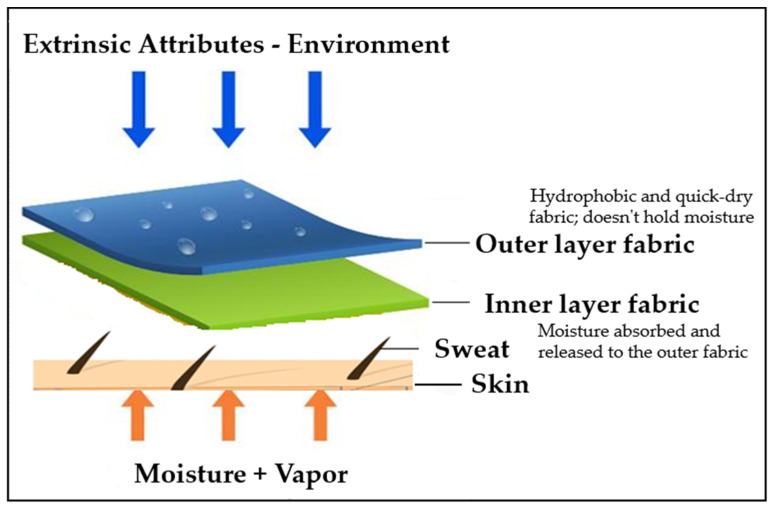
Bi-layer fabric construction.

Udaya Krithika et al. explored the moisture management capabilities of bi-layer knitted fabrics knitted using the same or various mixtures of cotton and polypropylene yarns. The face and reverse surfaces of micro-denier polyester and polyester staple fiber were investigated. The moisture management qualities of the fabrics created which determined the warm-cool feeling were evaluated, and the micro-denier polyester (inner) and the micro-denier polyester (outer) fabric had a higher moisture management property, offering high levels of comfort; it was recommended for summer, active, and sportswear, according to the findings [7]. Figure 1 shows the construction methods of bi-layer sportswear fabrics. The combination of layers can use adhesive bonding or stitching. Bonded layers provide high tensile strength, and bonds have the potential advantage of design flexibility. However, in terms of operating costs and total processing time that may be used to earn a profit, toxicity stitching is more effective than bonding [39].

Researchers found that a bi-layer knitted fabric with a one-tuck point made of bamboo had higher air permeability, thermal conductivity, water vapor permeability, wicking ability, and drying rate than other materials, while moisture absorption was reduced [40]. Synthetic material with good moisture transfer properties, such as polyester, nylon, acrylic, or polypropylene, is used on the inside of a multiple-layer textile, whereas a material that is a good absorbent of moisture (e.g., cotton, wool, viscose rayon, or their blends) can be used on the outside [2,41,42,43]. According to Thangamuthu et al., when using a double-layer fabric, the inner layer that comes into contact with the skin should be made of synthetic materials with good moisture-transfer qualities, such as polyester, acrylic, nylon, and polypropylene. Materials with strong moisture absorption capabilities, such as cotton, wool, viscose, or their blends, are suggested for the outer layer [6,44].

According to the literature review, the most popular choice for sportswear is a combination of several layers. Earlier research, on the other hand, only looked at a small number of fiber blended textiles. The impact of fabric structure and the order in which sports fabric layers are mixed on garment comfort have not been investigated. In recent years, many sportswear manufacturers have begun producing numerous varieties of sport layers with a range of fabric properties. However, previous research has not fully addressed these developments to determine the improved quality of existing sports clothing. The aim of this study is to investigate the effect of cloth layering in garments and their maintenance on the thermal comfort of sport fabrics. Sport fabric layer combinations and their effects are also within the scope of this research. Sportswear, thermal conductivity, thermal absorptivity, thermal resistivity, peak heat flow density ratio, stationary heat flow density, and related parameters including water vapor permeability resistance were evaluated and analyzed.

## 2. Materials and Methods

### 2.1. Materials

Sportswear manufacturers were asked to provide three types of interiors and four types of exterior sportswear layers from Pertex (Wichita Falls, TX, USA); and from the Czech companies O’Style, Nordblanc, Husky, and Altisport (Liberec, Czech Republic). All fabrics are commercially available and have been used in real-world applications. Twelve sets of samples were made using a combination of inner and outer layer fabrics to assess thermal properties. The layers for sport fabric were stitched together for the laboratory test. The structural properties of inner and outer layer fabrics are provided in Table 1. Sport fabric with 90% polyester/10% elastane, 100% polyester, 60% cotton/30% polyester/10% elastane, 90% polyester/10% spandex, and 100% polyamide with polyurethane laminated fabrics with woven and knitted structures were used for this study.

### 2.2. Methods

Alambeta^®^ equipment was used to measure the fabric thickness, thermal conductivity, thermal resistance, and thermal absorptivity of the samples. Low-temperature settings were used for the thermal behaviors, and a contact area of 120 mm^2^ was used for the tests. The water vapor resistance (Ret) property was measured using Permetest^®^ (skin model) equipment according to the ISO 11092 test method. The specimens were conditioned for 24 h at 20 ± 1 °C and 45 ± 3% relative humidity before being tested. Before the combination of outer and inner layers, thermal properties were measured for individual layers and are presented in Table 2.

#### 2.2.1. Fabric Thickness

The thickness of the layers was measured using the Alambeta^®^ testing device, and ten tests were carried out for each fabric type. The average fabric thickness is given in Table 2.

#### 2.2.2. Thermal Resistance

Thermal resistance R (×10^−3^) (K·m^2^ W^−1^) is a material’s ability to resist heat flow, and is the inverse of heat conductance. The R-value is a measure of how well heat can pass through a particular thickness of sportswear. 

#### 2.2.3. Thermal Conductivity

Thermal conductivity λ (×10^−3^) (Wm^−1^ K^−1^) is critical in determining how much heat moves through garments. An object’s thermal conductivity is the opposite of its thermal resistance. Alambeta^®^ equipment was used to determine the thermal conductivity. Thermal conductivity is a thermophysical measure of how much heat is transferred by conductive heat transfer through textiles. 

#### 2.2.4. Thermal Absorptivity

Thermal absorptivity (W·m^2^·S^1/2^·K^−1^) is the target estimation of the warm–cool sensation of textiles. The main impression is a warm–cool tendency. Heat exchange occurs between the skin and the clothing when a human body comes into contact with an article of clothing that is at a different temperature than the skin. If the thermal absorptivity of the apparel is high, it will provide a cooler feeling on the first contact [45].

#### 2.2.5. Water Vapor Resistance

The resistance of cloth to allow water vapor to pass through it is referred to as water vapor resistance. Water vapor resistance, or Ret, is the “inverse” of breathability, and is defined as the “water-vapor pressure differences of the two essences of a material separated by the resultant evaporative heat transfer per unit area toward the inclination,” according to the ISO 11092 standard.

## 3. Results and Discussion

In this section the experimental results are presented for single layers’ physical properties such as material type, thread density, fabric weight, and fabric structure. The developed sportswear’s thermo-physiological comfort properties and their interpretation are also illustrated.

### 3.1. Thermal Resistance

**Figure 2:**
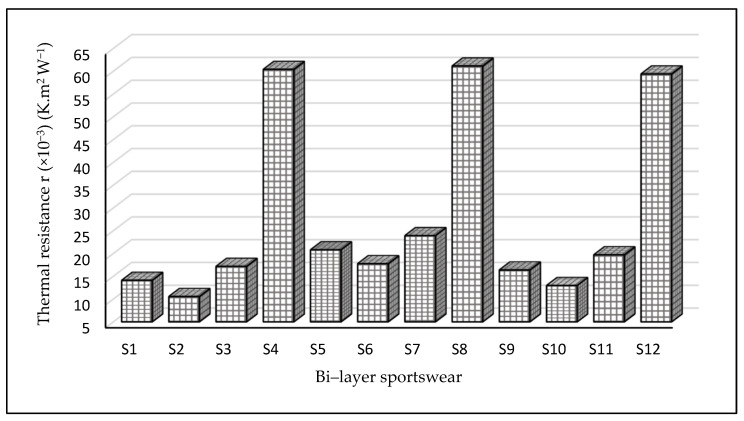
Thermal resistance of sportswear. The thermal resistance is a measurement of a material’s ability to resist heat flow. Samples S8, S4, and S12 recorded the highest thermal resistance values, respectively, while S2 documented the lowest thermal resistance.

As can be seen in Figure 2, sportswear (S8) made from IL2 + OL4 had the highest thermal resistance of 61.16 (×10^−3^) (K·m^2^ W^−1^), followed by samples S4 with a value of 60.41 and S12 with a value of 59.41(×10^−3^) (K·m^2^ W^−1^). On the other hand, samples S1, S10, and S2 had a lower thermal resistance of 14.11, 12.98, and 10.56 (×10^−3^) (K·m^2^ W^−1^), respectively. This is because thicker fabrics contribute to greater resistance (see Table 2), and sportswear made from a combination of 100% elastane and 100% polyester had lower thermal conductivity as compared to sportswear made from polyamide. Polyester also has a high level of heat resistance and thermal stability. Because of its minimal moisture absorption, easy care qualities, and low cost, polyester fiber is most widely used in base textiles for activewear. Polyester is hydrophobic, which means it does not absorb water. The majority of polyester used in base layer apparel, on the other hand, has been chemically treated to allow it to wick moisture [41]. Therefore, sportswear with higher thermal resistance is suitable for the cold season.

Furthermore, the statistical results show that the thermal resistance of bi-layered fabrics was significantly different at a *p*-value of 0.000 and a significance level of α = 0.05. If the *p*-value is greater than 0.05, we considered it to mean that the samples had similar properties; conversely, a *p*-value less than 0.05 implied a significant difference in their properties (see Table 3). On the other hand, if F (F calculated) was greater than F crit (F critical), it indicates that the samples had different properties.

### 3.2. Thermal Conductivity

**Figure 3:**
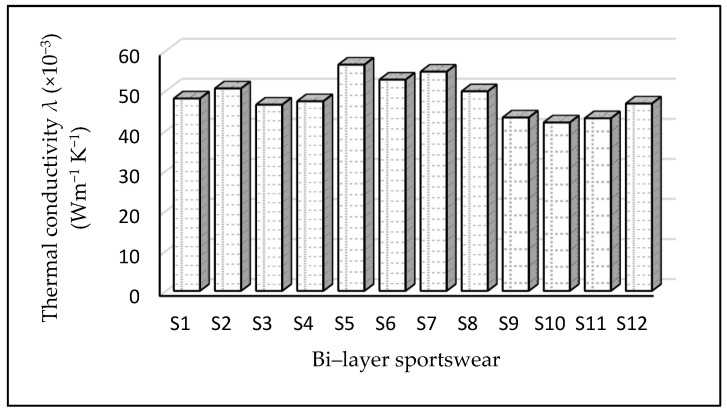
Thermal conductivity of sportswear. Thermal conductivity is the capability of a material (bi-layer sportswear in this study) to conduct heat, and it represents the quantity of thermal energy that flows per unit time through a unit area. Sample S5 recorded the highest while S10 recorded the lowest result.

Thermal conductivity is a complex attribute of athletic clothing that reflects its ability to conduct heat. Heavy sweat is created in the body as a result of heat dissipation, resulting in an accumulation of moisture on the skin. Sample S5 (56.47 × 10^−3^ (Wm^−1^ K^−1^)) showed the highest thermal conductivity followed by S7 (54.72 × 10^−3^ (Wm^−1^ K^−1^)), and other results are shown in Figure 3. This can be explained by the fact that the thickness of S5 was lower (see Table 2) than that of all other bi-layer sportswear evaluated here. The second reason is that sample S5 was made from an inner fabric composed of 60% cotton/30% polyester/10% elastane and an outer fabric 100% polyamide with a PU coating. Therefore, samples S9, S10, and S11 had lower thermal conductivity because of the PU coating trapped in the fabric structure which provided a lower thermal conductivity. Polyamide-based fabrics had a slightly higher thermal conductivity, while cotton-based fabric combinations had the lowest thermal resistance due to higher thermal conductivity. The combination of a 100% elastane inner layer with an outer fabric of polyamide fabrics led to a slight decrease in thermal conductivity as compared to others. Polyester has a lower thermal conductivity, and while considering the thermal properties of textile fabrics, the determining factor is the thermal properties of the polyester fabrics. In an earlier study by Das et al. [42], comparable conclusions were reached. It is also evident that as stitch density drops, thermal conductivity reduces. The thermal conductivity of bi-layer athletic fabrics is largely determined by geometric parameters such as thickness, stitch density, and areal density/mass per unit area of the fabric.

As evident in Table 3, the 12 samples were completely different in terms of thermal conductivity (*p*-value = 0.000).

### 3.3. Thermal Absorptivity

**Figure 4:**
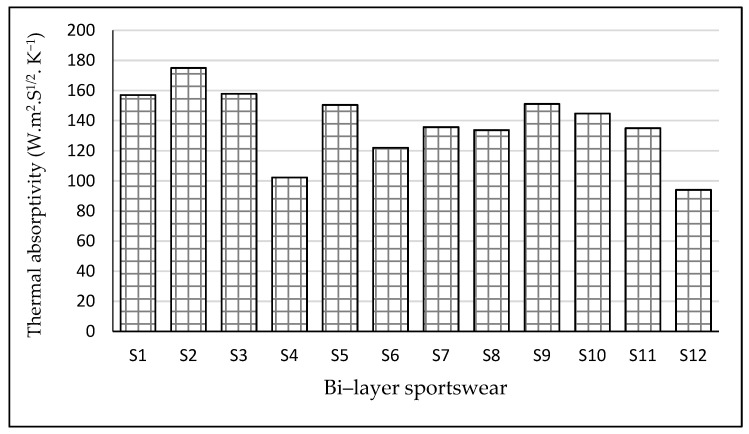
Thermal absorptivity of sportswear. Thermal absorptivity is the quantity of heat penetrating a sportswear fabric during the time period when the temperature is raised rapidly. This measure indicates the warm–cool feelings of the sportswear.

The thermal absorptivity (W·m^2^·S^1/2^·K^−1^) is a measurement of how heat is absorbed by textile materials at a specific wavelength. Thermal absorption is a surface attribute that can be used to analyze the fabric’s properties in terms of its “cool–warm” feeling. Fabrics that have a low thermal absorption value make us feel ‘warm. Figure 4 shows that the value of thermal absorptivity increased as fabric thickness decreased. Sportswear samples S6, S4, and S12 had much lower values of thermal absorption of 122, 102.3, and 94.08 W·m^2^·S1/2·K^−1^, respectively, suggesting that they are suitable for cold weather and can give a warmer feeling to the wearer. On the contrary, sportswear prepared from a 90% polyester/10% elastane inner layer and a 100% polyamide outer had the highest values of thermal absorptivity, 157, 157.8, and 175 W·m^2^·S1/2·K^−1^ for samples S1, S2, and S3, respectively. This is most likely the effect of the textile fabric’s lamination and thickness. The thermal absorptivity of sportswear depends upon the thermal conductivity and limit of the texture, as well as the contact zone of the skin and texture surface. As seen from the results in Figure 4, the thermal absorptivity of sportswear fabrics increased as the thermal conductivity of fabrics increased. Sportswear with a high thermal absorptivity is appropriate for hot weather, making it excellent for summer clothing. Coated fabrics, in both circumstances, had the highest absorption. In light of the above findings, it is feasible to deduce that material with a regular, flat, and smooth surface feel cooler than fabrics with less regularity and smoothness, as well as higher surface roughness.

As can be seen in Table 3, the statistical analysis showed that the thermal absorptivity of sportswear was significantly different between different samples (*p*-value = 0.011).

### 3.4. Ratio of Maximum and Stationary Heat Flow Density

**Figure 5:**
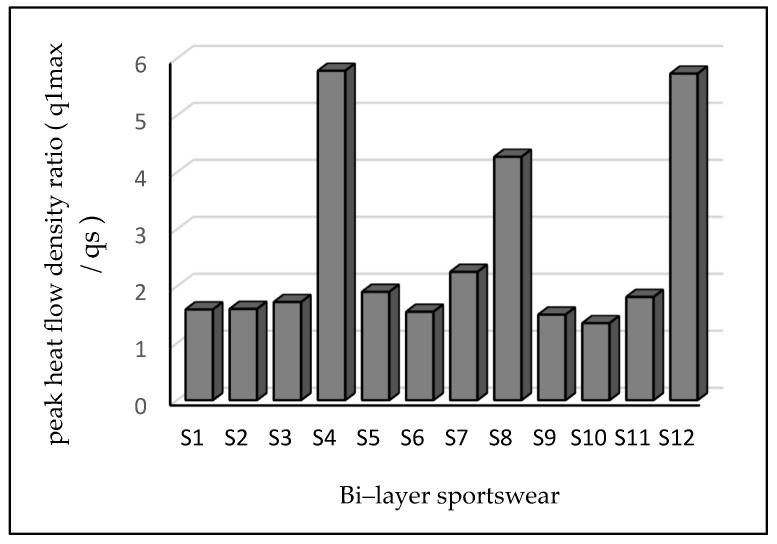
Ratio of maximum and stationary heat flow q_max_/q_s_.

The maximum heat flow density from the skin to the fabric occurs when a cold fabric touches human flesh. The heat flow eventually stabilizes at a predetermined level qs, which is referred to as the stationary heat flow density. The ratio of maximum to stationary heat flow density for samples S8, S12, and S4 had higher values of 4.27, 5.73, and 5.78 qm/qs, respectively. These results showed a similar trend with thermal resistance results. Sample S4 was made from a 90% polyester/10% elastane inner layer with a 100% polyester coated outer fabric. The second highest value of the ratio of maximum to stationary heat flow density was recorded in sample S12, was made from a 60% cotton/30% polyester/10% elastane inner layer and a 100% polyester coated outer layer. Generally, bi-layered fabrics based on PU-coated polyester showed a higher value than others. This is because the ratio of maximum to stationary heat flow density is affected by fabric thickness and the upper surface of fabrics. On the other hand, sportswear made from 90% polyester/10% elastane inner layer and a PU-coated 100% polyamide outer layer had a lower ratio of maximum to stationary heat flow density. Similar concepts reported by previous researchers showed elastomeric finishing fabrics had a higher ratio [43]. The maximum heat flow is one of the factors that characterize fabric thermal insulation, and it is a surface attribute analogous to thermal absorption [44]. Therefore, the research findings presented in Figure 5 confirm the ideal concept. Table 3 shows that the ratio of maximum and stationary heat flow of the sportswear had a significant difference, at a *p*-value of 0.000 at α = 0.05.

### 3.5. Stationary Heat Flow

The stationary heat flow density qs is defined by Equation (1):

(1) $$q_{s}=\frac{Q}{F.\;\tau }\;,\;{\mathrm{Wm}}^{-2}$$

where:

Q is the amount of heat;

F is the area through which the heat is conducted; and

Τ is the time of flow.

**Figure 6:**
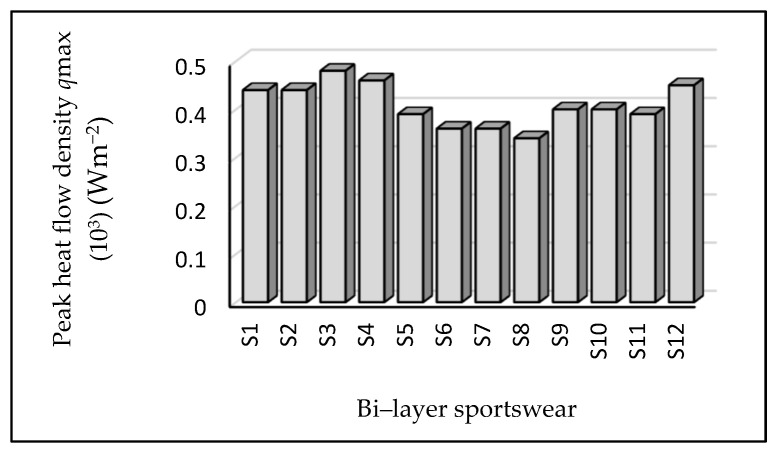
Stationary heat flow density. Sample S3 had a higher value of stationary heat flow density, indicating that when the percentage of polyester increases, the stationary heat flow density increases proportionally. This indicates that hydrophobicity affects heat flow.

As shown in Figure 6, sample S3 had the highest value of stationary heat flow density of 0.48 Wm^−2^ followed by samples S4 (0.46) and S12 (0.45). On the contrary, samples S8, S7, S6, and S5, which were made of a 60% cotton/30% polyester/10% elastane inner layer fabric and four types of outer layers (Table 1), had lower stationary heat flow densities.

### 3.6. Water Vapor Resistance

**Figure 7:**
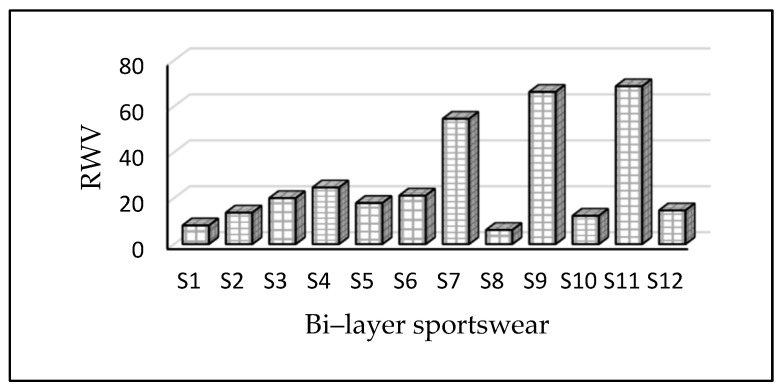
Water vapor resistance of sportswear. Sportswear fabric made from polyamide and polyester fabric prevents the passage of water more than the one made from cotton fabrics (see S7, S9, and S11).

When there is very little sweating or insensible perspiration, water vapor permeability is particularly essential [44]. To eliminate wetness on the skin, the clothing should be able to release moisture vapor trapped in the microclimate to the atmosphere [45]. To feel comfortable during strenuous activity when liquid sweat generation is strong, clothing should have good liquid transfer properties [46]. Water vapor permeability is an important feature for badminton clothing textiles. Perspiration is created when the human body is overheated, and the body heat evaporates it [46]. The higher the RWVP, the lower the Ret, and the better the thermal comfort of the garment. Breathability is measured by determining resistance to evaporative heat transfer (RET). The lower the RET value, the higher the fabric’s breathability. In this investigation, the results are presented in Ret. As seen in Figure 7, samples S7, S9, and S11 sportswear had the highest value of water vapor resistance of 54.55, 66.25, and 68.75%. This means they will provide less comfort and poor breathability for the wearer as compared to other fabrics. The variations of water vapor permeability are most probably due to an increase in the thickness of fabric and coated fabric. In contrast, samples S12, S2, S10, S1, and S8 had lower water vapor resistances of 14.68, 13.7, 12.32, 8.12, and 6.12%, respectively. It was observed that sportswear with a 90% polyester/10% elastane inner layer showed lower water vapor resistance than sportswear with a 60% cotton/30% polyester/10% elastane or 100% elastane layer. As a result, these athletic garments have a higher level of breathability and can give excellent comfort to the wearer. We determined that the water vapor permeability of bi-layer fabrics is influenced by geometric characteristics and their material nature. Moreover, the water vapor permeabilities of the studied sportswear were significantly different at a *p*-value of 0.000.

## 4. Conclusions

This research work aimed to investigate the effect of layered fabrics in sportswear garments and their maintenance of the thermal comfort of the wearer. Twelve sportswear fabrics were developed by stitching using a combination of three inner- and four outer-layer fabrics, and their thermal resistance, conductivity, absorption, ratio of maximum to stationary heat flow density, stationary heat flow density, and water vapor resistance were evaluated. Sportswear (S8) made from a 60% cotton/30% polyester/10% elastane inner layer and a 100% polyester outer layer had the maximal thermal resistance of 61.16 (×10^3^ K·m^2^ W^−1^). Fabrics made of polyamide had a slightly higher thermal conductivity, whereas cotton-based fabric combinations had the lowest thermal resistance due to their higher thermal conductivity. In contrast, the combination of a 100% elastane inner layer with a polyamide outer fabric led to a small decline in thermal conductivity. As the fabric thickness decreased, the value of thermal absorptivity increased. Sportswear codes S6, S4, and S12 exhibited substantially lower thermal absorption values of 122, 102.3, and 94.08 W·m^2^·S^1/2^·K^1^, respectively, and are suited to cold weather. Water vapor resistance was lower in sportswear with a 90% polyester/elastane10% inner layer than in sportswear with a 60% cotton/30% polyester/10% elastane or a 100% elastane inner layer. As a result, these sportswear garments have a higher level of breathability and will provide excellent comfort to the wearer. We conclude that the geometric properties and the nature of the material affects the water vapor permeability of bi-layered sportswear. Generally, according to the results, sportswear with a 60% cotton/30% polyester/10% elastane inner layer and a PU-coated 100% polyamide outer layer is recommended for the winter season. Sportswear made of a 90% polyester/10% elastane or 100% elastane inner layer, with an outer layer of 100% polyamide fabric, had lower water resistance and is suitable for summer weather.

## Figures and Tables

**Table 1:** Characteristics of sport fabric layers.

Layers	Single Layer Code	Type of Material	Pattern/Printed	Thread Density/Cm		Fabric Weight (g/m^2^)	Fabric Structure
				Warp/Wales	Weft/Course		
Inner layer	IL1	90% polyester 10% elastane	dyed	30	17	177	Woven ripstop
	IL2	60% cotton 30% polyester 10% elastane	bleached	29	16	198	Woven ripstop
	IL3	100% elastane	white	21	10	167	Woven ripstop
Outer layer	OL1	100% polyamide	dyed	14	22	126	Plain knitted PU coated
	OL2	100% polyamide	dyed	24	48	59	Plain knitted PU coated
	OL3	95% polyester 5% elastane	dyed	15	22	160	Plain knitted
	OL4	100% polyester	printed	26	18	361	Plain woven

**Table 2:** Thermal characteristics of single sport cloth layers.

**Single Layer Code**	**Thickness (mm)**	**Thermal Resistance**	**Thermal Conductivity**	**Thermal Absorptivity**	**Peak Heat**	**Water Vapor Resistance**
IL1	53.12	7.25	51.12	168	0.52	1.87
IL2	40.35	13.58	44.46	141	0.39	3.48
IL3	40.13	9.11	40.26	135	0.43	1.86
OL1	30.63	6.17	30.67	11	0.51	73.35
OL2	21.12	2.87	20.26	181	0.67	15.58
OL3	31.45	8.98	31.45	131	0.41	3.39
OL4	40.76	49.51	40.78	71	0.15	86.26
**Combined Fabric Code**	**Prepared from Inner Layer (IL) and Outer Layer (OL)**	**Combined Fabric Code**	**Prepared from Inner Layer (IL) and Outer Layer (OL)**			
S1	IL1 + OL1	S7	IL2 + OL3			
S2	IL1 + OL2	S8	IL2 + OL4			
S3	IL1 + OL3	S9	IL3 + OL1			
S4	IL1 + OL4	S10	IL3 + OL2			
S5	IL2 + OL1	S11	IL3 + OL3			
S6	IL2 + OL2	S12	IL3 + OL4			

**Table 3:** Statistical analysis of variance thermo-physiological comfort.

Properties	Source of Variation	SS	df	MS	F	*p*-Value	F Crit
Thermal resistance	Between Groups	43,649.33	11	3968.121	9766.801	0.000	1.8784
	Within Groups	43.87896	108	0.406287			
Thermal conductivity	Between Groups	2329.853	11	211.8048	57,187,299	0.000	1.8783
	Within Groups	0.0004	108	0.0000			
Thermal absorptivity	Between Groups	286,145.4	11	26,013.22	2.590388	0.011	1.8784
	Within Groups	1,766,504	108	16,356.52			
Peak heat flow density (q1max)	Between Groups	0.175427	11	0.015948	83.12601	0.000	1.8784
	Within Groups	0.02072	108	0.000192			
Peak heat flow density ratio (q1max/qs)	Between Groups	305.8382	11	27.80348	236,625.3	0.000	1.8783
	Within Groups	0.01269	108	0.000118			
Water vapor resistance	Between Groups	49,967.29	11	4542.481	70,776.33	0.000	1.8898
	Within Groups	6.161356	108	0.064181			

SS—sum square, df—degree of freedom.

## Data Availability

All data have been included in the manuscript.
